# X-linked SCID with a rare mutation

**DOI:** 10.1186/s13223-021-00605-7

**Published:** 2021-10-11

**Authors:** Fatemeh Sadat Mahdavi, Mohammad Keramatipour, Sarina Ansari, Samin Sharafian, Arezou Karamzade, Marzieh Tavakol

**Affiliations:** 1 Student Research Committee, Alborz University of Medical Sciences, Karaj, Iran; 2 Department of Medical Genetics, School of Medicine, Tehran University of Medical Sciences, Tehran, Iran; 3 Department of Allergy and Clinical Immunology, Mofid Children’s Hospital, Shaheed Beheshti University of Medical Sciences, Bushehr, Iran; 4 Department of Medical Genetics, School of Medicine, International Campus, Tehran University of Medical Sciences, Tehran, Iran; 5 Non-Communicable Diseases Research Center, Alborz University of Medical Sciences, Karaj, Iran

**Keywords:** Severe combined immunodeficiency, IL2RG gene, Immunodeficiency, Primary immunodeficiency disorders, γc mutation

## Abstract

**Background:**

Severe combined immunodeficiency (SCID) is a group of relatively rare primary immunodeficiency disorders (PIDs), characterized by disturbed development of T cells and B cells, caused by several genetic mutations that bring on different clinical presentations. SCID may be inherited as an autosomal recessive or an X-linked genetic trait.

**Case presentation:**

A 6-year-old male presented with a history of food allergy, productive coughs, and recurrent purulent rhinitis, poor weight gain and hypothyroidism. The total count of CD4+ T lymphocytes, along with their naïve and central memory subpopulations, as well as central memory CD8+ T cells were decreased in flow cytometry. A nucleotide substitution in exon one of interleukin 2 receptor gamma chain (IL-2RG) gene (c.115 G>A, p.D39N, ChrX: 70,331,275) was reported, based on which the diagnosis of X-liked SCID was confirmed. Antiviral and antibiotic prophylaxis, along with monthly IVIG (intravenous immunoglobulin) was started and the patient was subsequently referred for hematopoietic stem cell transplantation.

**Conclusion:**

PIDs should be considered as the differential diagnosis in any patient with unexplained and bizarre symptoms associated with recurrent infections, allergic and autoimmune manifestations. Clinicians should also bear X-SCID in mind in case of approach to any patient with poor weight gain, unusual allergic or endocrine manifestations, even in the case of a normal or increased level of serum immunoglobulins or T and B cells numbers.

## Background

Severe combined immunodeficiency (SCID) consists of a heterogeneous group of heritable defects characterized by serious impairment of cellular and humoral immune systems due to a defect in T-cells development [1]. Neonates with SCID are usually normal at birth, often remain undiagnosed until life-threatening infections happen [2, 3]. The most common form of SCID is X-linked SCID (X-SCID), which accounts for 50–60% of cases, characterized by the complete deficiency of mature T and NK lymphocytes and a normal or moderately increased number of B cells with a typical phenotype [4].

Interleukin-2 receptor (IL-2R) is made from three parts including IL-2Rα and IL-2Rβ and IL-2Rγ. The IL2RG gene encodes the γc portion and mutations in the IL2RG gene can cause X-SCID [5]. Not only does the common γc portion interact with the IL-2 cytokine, but it is also shared by other leukocyte cytokine receptors (IL-4, IL-7, IL-9, IL-15, and IL-21). IL-2 has a fundamental role in the development of regulatory T cells (Tregs), which are crucial in maintaining peripheral tolerance. Moreover, it is involved in the elevation of NK cells’ cytolytic activity as well as signaling pathways of various other cytokines [6]. The lack of a functional γc gene, hence, leads to the blockage of multiple cytokine pathways. Early lymphoid progenitor cells, consequently, will be unable to respond to the signals of the above-mentioned interleukins, which are essential cytokines for the normal development and function of T cells, NK cells, and also the late stages of B cells development [7]. Genetic analysis is the golden standard for definitive diagnosis. However, it has been recently suggested that in case of high suspicion of the diagnosis, performing functional assessment and γc signaling would be helpful if unknown variants of the gene are reported [8].

Hematopoietic stem cell transplantation (HSCT) is considered the curative management of this disorder. Gene therapy has also been proposed as an alternative therapeutic approach with a high rate of success in X-SCID. The only concern is the oncogenic capacity of this process [9]. Typical X-SCID, not promptly corrected with HSCT or gene therapy, could be fatal. However, untreated patients with hypomorphic variants mutations of the IL2RG gene may survive longer and present with autoimmune in addition to infectious complications [10]. Herein, a rare mutation in the IL2RG gene is described in a patient presented with allergic and infectious disorders, along with decreased number of CD4+ T cells, naïve and central memory CD4+ T cells.

## Case presentation

A 6-year-old male was hospitalized due to persistent productive coughs, otalgia, and recurrent persistent purulent rhinorrhea without complaint of chest tightness or breathlessness. He was the only child of the family whose parents were second cousins with no report of allergic or immunodeficiency disorders in the family members. There was a history of frequent hospital admissions since the age of 7 months with a variety of complaints including allergic and infectious problems (Table 1). He was diagnosed with cow’s milk protein allergy at the age of 15 months. A strict cow’s milk-free diet was followed until his second birthday when his diarrhea stopped and never recurred.

**Table 1:** Summary of the patient’s clinical manifestations

	Clinical presentations	Age of presentation	Paraclinical findings
Allergy	Food allergy presenting with chronic and bloody diarrhea	Starting at 12 months disappearing at 24 months	Stool exam WBC: many (NR: 0–5/HPF) RBC: 30–35 (NR: 0–5/HPF) Stool α1 antitrypsin: 125 mg/dl (NR: < 54 mg/dl) calprotectin: 850 mcg/g (NR: < 50 mcg/g) SPT^Ɨ^: significantly positive for pistachio, egg white, wheat flour, ray flour, and milk tTG IgG^±^: 1.3 IU/ml (Negative range: < 10 IU/ml) anti-gliadin IgG:10.4 U/ml (Negative range: < 12 IU/ml)
Infectious	Frequent upper and lower respiratory tract infections Recurrent otitis media Cervical lymphadenopathies Herpetic lesions on the hand fingers	emerging at 7 months continuing until a few months after starting of the IVIG therapy	Sputum sample PCR^$^: negative for EBV, HIV, meningococcus, pneumococcus, and influenza virus positive for haemophilus influenza on one occasion Anti-toxoplasma IgG and IgM antibodies: negative anti-cytomegalovirus (CMV) IgM antibody: negative

NR: Normal range

^Ɨ^Skin prick test using Inmunotek extracts (Inmunotek Co., LTD, Spain)

^±^IgG antibody to tissue transglutaminase

^$^Polymerase chain reaction

Medical history was negative for oral candidiasis, persistent ulcers, or abscesses. He was gone through extensive diagnostic workup during the preceding admissions since the age of 7 months. Regarding the patient’s TSH level of 5.6 mg/dl and failure to gain normal weight, injectable growth hormone plus oral levothyroxine was prescribed with the diagnosis of hypothyroidism, about 3 months before his last admission at the age of 6.

The most distinguished findings on physical examination were stunted growth, multiple anterior and posterior cervical, as well as submandibular lymphadenopathies in addition to diffuse crackles, heard in both lungs. The patient’s weight and height were below the 5th percentile for age and gender (age = 6 years old, weight = 17.5 kg, height = 103.5 cm, BMI = 15.9). Despite the history of multiple occasions of otorrhea, no active ear discharge or tympanic perforation was found at the age of 6. Although Patent Foramen Ovale (PFO) was reported in his earlier echocardiography, cardiac auscultation was normal. No hepatosplenomegaly or abdominal tenderness was detected.

**Fig. 1:**
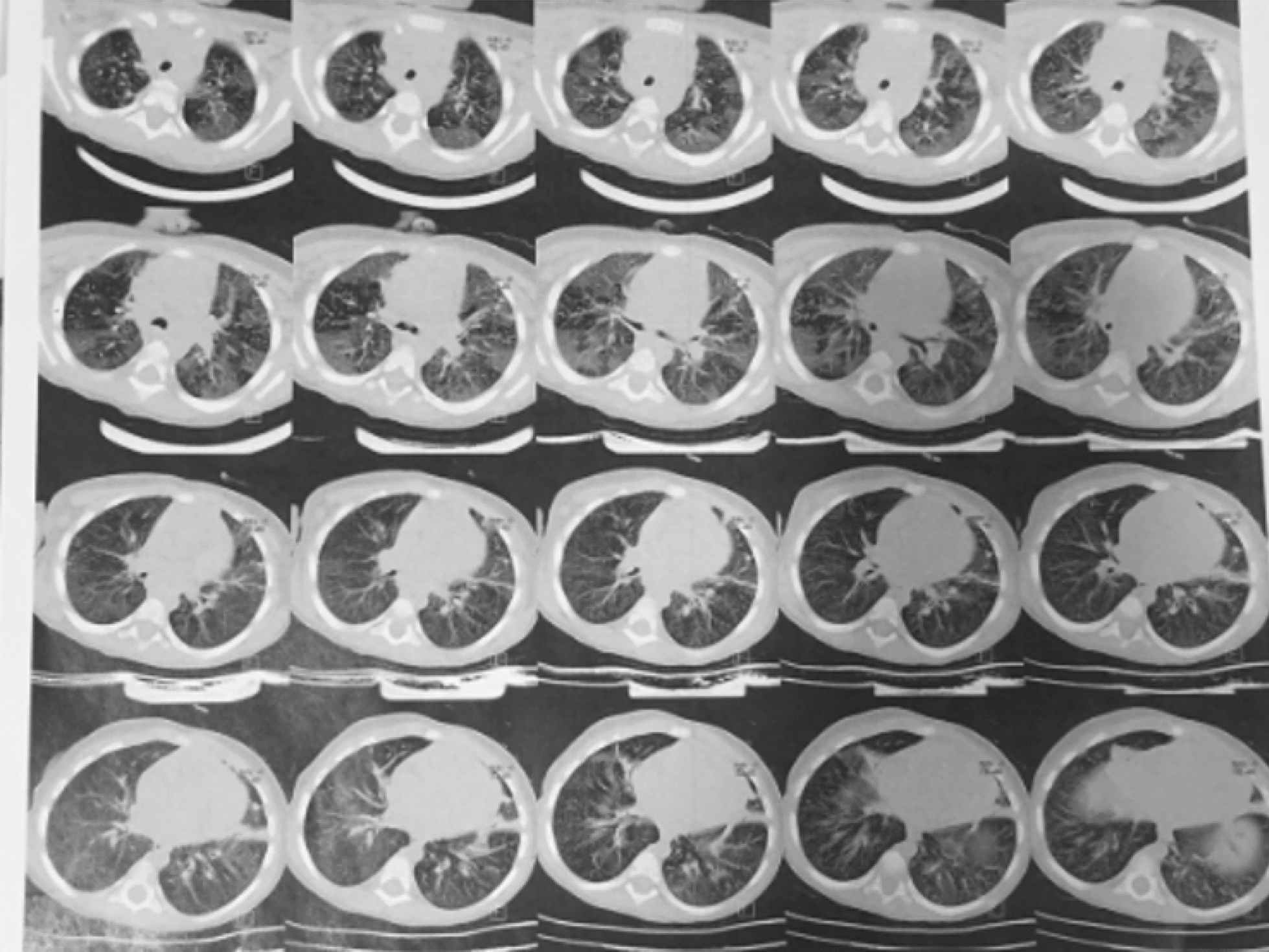
Lung CT scan

Ground glass opacity in basal segments of right lower lobe and patchy infiltration in addition to interlobular septal thickening in the peripheral zone of lower lobes of both lungs along with mediastinal and axillary lymph node hyperplasia were detected on chest computed tomography (CT) scan, performed at the age of 4. Consolidation, peripheral ground-glass opacity, and air bronchogram on both sides, as well as mild peribronchial thickening, were reported on chest CT scan at the age of 6, indicating pneumonia, without any signs of lymphadenopathy or pleural effusion (Fig. 1). Pansinusitis features including bilateral opacification of ethmoid and maxillary sinuses with membrane thickening and obstruction of the infundibulum were seen in the CT scan of the paranasal sinuses at the age of 6. Reactive nodes without any evidence of EBV infection were reported in the pathology of cervical lymph nodes after excisional biopsy.

**Fig. 2:**
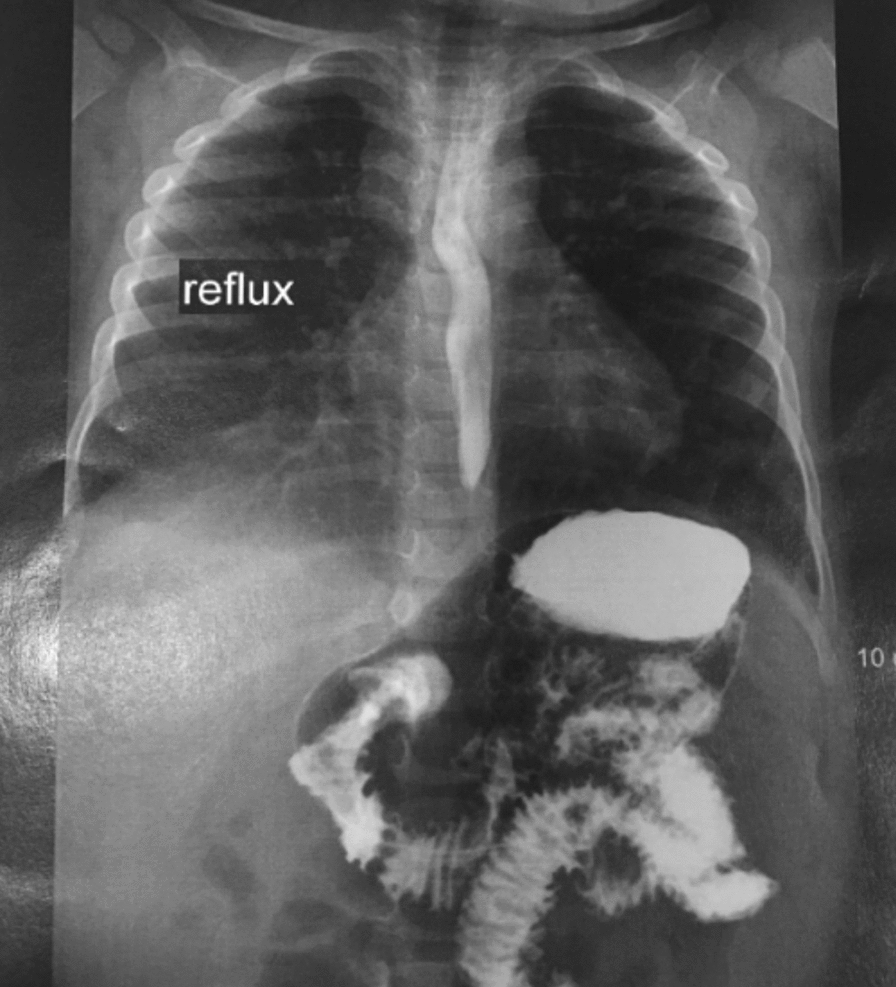
Suspicious image of a diverticulum in the left esophageal wall

**Fig. 3:**
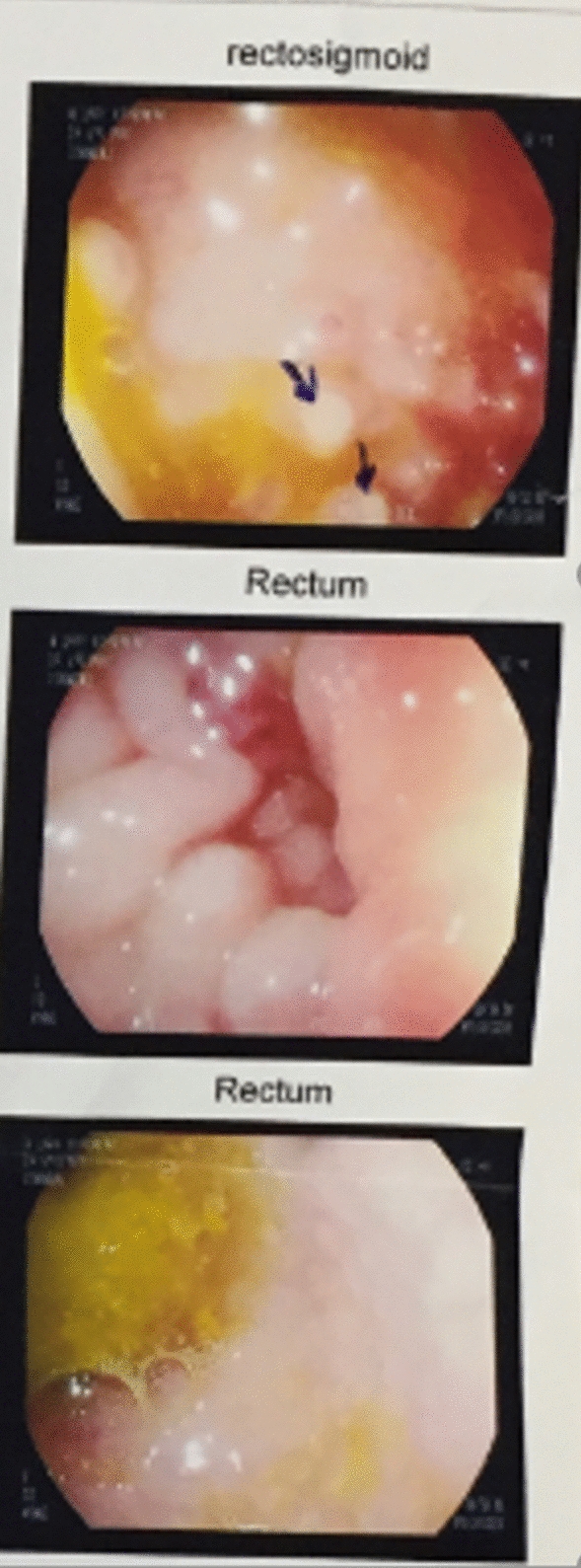
Hypernodularity with exudate in the rectum and sigmoid colon at colonoscopy

The gastrointestinal examination was carried out during the period of chronic diarrhea, before the second birthday. Esophageal diverticulum, gastroesophageal reflux, and regurgitation without any signs of hiatal hernia, pyloric stenosis, or intestinal obstruction were found in the barium swallow test showed in Fig. 2. Upper gastrointestinal endoscopy, at the age of 2, showed inflammation of the lower esophagus with hiatal hernia and accumulation of a great amount of food in the lower third of the esophagus, but the stomach reported being normal. Further workup including colonoscopy and rectosigmoid biopsy showed nodular lymphoid hyperplasia and exudate in the rectum, colon, and sigmoid in addition to inflammation and increased number of eosinophils in the lamina propria with active lymphoid follicles and active pancolitis, suggesting a form of inflammatory bowel diseases or allergic disorders (Fig. 3).

Further workup was performed to rule out the differential diagnosis of malignancies and immunodeficiencies. Complete blood count (CBC) showed hypochromic and microcytic anemia along with eosinophilia (Table 2). No significant abnormality was found in the assessment of serum electrolytes as well as liver and kidney function tests.

**Table 2:** Laboratory test findings in our patient

Test	4 years old	5 years old	6 years old	Normal range
Leukocytes (×1000/ul)	16.7	23.16	12.03	4–11
Lymphocyte (×1000/ul)	7.68	12.62	6.25	1.8–4.5
Neutrophil (×1000/ul)	7.84	7.17	3.96	4.5–7.5
Eosinophil (×1000/ul)	–	–	0.721	0.03–0.35
Monocyte (×1000/ul)	–	–	0.108	0.2–0.8
Hemoglobin (g/dl)	8.4	–	12.5	11.5–13.5
MCV (fL)	67.8	–	78.6	80–98
Platelet (×1000/ul)	499	–	271	150–450
IgA (mg/dl)	195	174	128	80–350
IgG (mg/dl)	1490	1760	1106	443–1095
IgM (mg/dl)	144	246	119	27–195
IgE (IU/ml)	117	109	53	100–200
Antitetanus (IU/ml)	0.04	0.1	–	> 0.15
Antidiphteria (IU/ml)	0.01	0.06	–	> 0.15
Antipneumoccoc (ug/ml)	6.2 (before vaccination)	56 (after vaccination)	–	≥ 640 (after vaccination)
T3 (ng/ml)	1.9	–	–	0.9–2.2
TSH (ml/dl)	2.3	–	5.6	0.85–6.5
FT4 (ng/ml)	1.3	–	-	0.8–2

MCV: Mean Corpuscular Volume, Ig: Immunoglobulin, TSH: Thyroid Stimulating Hormone, FT4: Free T4

Immunologic evaluation (illustrated in Tables 2, 3, 4) revealed hypergammaglobulinemia. Despite receiving routine DPT (tetanus, diphtheria, and pertussis) vaccination, anti-tetanus toxoid IgG and anti-diphtheria toxoid antibody were reported to be lower than protective level. Considering the delayed diagnosis, he received MMRV (measles, rubella, and mumps vaccine) at the age of 12 and 18 months in the context of routine vaccination and surprisingly tolerated both doses. Moreover, anti-pneumococcal antibody levels were lower than normal both before and after vaccination. Purified protein derivative (PPD) skin test was non-reactive despite receiving Bacillus Calmette–Guérin (BCG) vaccine at birth. NBT (Nitroblue Tetrazolium) test result was within normal limits. Cystic fibrosis was ruled out owing to the negative result of the sweat chloride test (PR: 28 mmol/l, NR: < 30 mmol/l). Other general laboratory test findings are summarized in Table 2. Flow cytometric lymphocyte subsets enumeration (Tables 2 and 3) showed a decreased total count of CD4+ T lymphocytes and their subpopulations as well as CD8+ T cells. Other subsets of lymphocytes were within normal ranges. The patient’s stimulation index to BCG and candida not PHA (Phytohemagglutinin) in lymphocyte transformation test (LTT), done using BrdU cell proliferation assay Elisa Kit, was lower than the normal range (Table 4). Lymphocyte proliferation was also assessed by carboxyfluorescein succinimidyl ester (CFSE) test, in which gradual halving of CFSE fluorescence within daughter cells after cell divisions is evaluated. The proliferation index is the average number of divisions of just the responding cells (cells that underwent at least one division) and the division index is the average number of divisions for all of the cells in the original starting population [11]. The patient's division index was 0.6 (NR > 0.4) and proliferation index was 1.5 (NR > 1.3), both within normal limits.

**Table 3:** Lymphocyte subsets results in flow cytometry

Normal range	6 years old	5 years old	4 years old	Test
65–88% (1400–3700)	64 (4000)	–	60 (4600/µl)	CD3% Number (cells//µl)
26–62% 700–2200	17.5% (1093/µl)	17% (2450/µl)	19% (1459/µl)	CD4%
14–44% 490–1300	34.5% (2156/µl)	31% (3930/µl)	27% (2073/µl)	CD8%
0.9–2.9	0.5	0.5	0.7	CD4/CD8
	1.98% (borderline) Absolute count = 125	–	–	TCRαβ + CD4-CD8-%
2–27% 390–1400	13.3% (8312/µl)	23% (2900/µl)	26.2% (2012/µl)	CD19%
14% 390–1400	13.5% (843/µl)	25% (3155/µl)	23.7% (1820/µl)	CD20%
9%	21% (16 + 56 +) (1312/µl)	19% (2397/µl)	26.6% (2042/µl)	CD16%
12%	24% (1500/µl)	21% (2650/µl)	26.2% (2012/µl)	CD56%
32–76%	81.5%	–	–	Naïve B cell
1.2–10%	2.84%	–	–	Marginal zone like B cells
2–16%	2.78%	–	–	Switched memory B cells
1–12%	12%	–	–	Transitional B cells
1–4%	4%	–	–	CD21 B cell
0.2–4%	0.4%	–	–	Plasma cell
32–71%	23.6%	–	–	Naïve CD4 T cell
10–39%	5.1%	–	–	Central memory CD4 T cell
9–39%	45.5%	–	–	Effector memory CD4 T cell
19–76%	22.5%	–	–	Naïve CD8 T cell
0.3–9%	0.06%	–	–	Central memory CD8 T cell
6–33%	36.3%	–	–	Effector memory CD8 T cell

**Table 4:** Lymphocyte Transformation Test (LTT) Results

Mitogen/ Antigen	Patient’s stimulation index	Control’s stimulation index	Normal range
PHA	4.2	5	≥ 3
BCG	2.2	4.6	≥ 2.5
Candida	1.8	3.9	≥ 2.5

## Genetic investigation

In chromosome analysis, the patient showed a normal male karyotype (46, XY). Then blood samples were collected from the patient and his parents in EDTA-containing tubes. Genomic DNA was extracted from whole blood using a Blood SV-mini kit (GeneAll Biotechnology Co., LTD, South Korea) according to the manufacturer’s instruction. The concentration and purity of DNA were assessed before performing whole-exome sequencing (WES).

Library preparation was performed using Twist human core exome plus kit (Twist Bioscience, USA) using manufacturer instruction. Sequencing of libraries was done by high-throughput paired-end sequencing using the NovaSeq platform (Illumina Inc., CA, USA).

Sequencing short reads were aligned to the reference human genome hg19 from the UCSC genome browser (University of California, Santa Cruz, USA) via the Burrows-Wheeler Aligner (BWA) program. Variant calling was done using the Genome Analysis Toolkit (GATK). Detected variants were annotated using appropriate databases.

Proper filtering and then the interpretation of a shortlist of variants in terms of pathogenicity was performed based on ACMG (American College of Medical Genetics and Genomics) guideline for variant interpretation.

The potential impact of a given variant on the function or structure of the encoded protein was analyzed to evaluate the pathogenicity of the novel variants. The analysis was carried out based on conservation, physical properties of the amino acids, or possible occurrence in regulatory or splicing motifs using bioinformatics tools. OMIM, PubMed, and Human Gene Mutation Databases (HGMD) were reviewed for previous publications related to the candidate causative gene.

The causative variant, detected by WES, was validated in the patient and his mother using Polymerase Chain Reaction, followed by Sanger sequencing (PCR-Sanger sequencing).

## Results

**Fig. 4:**
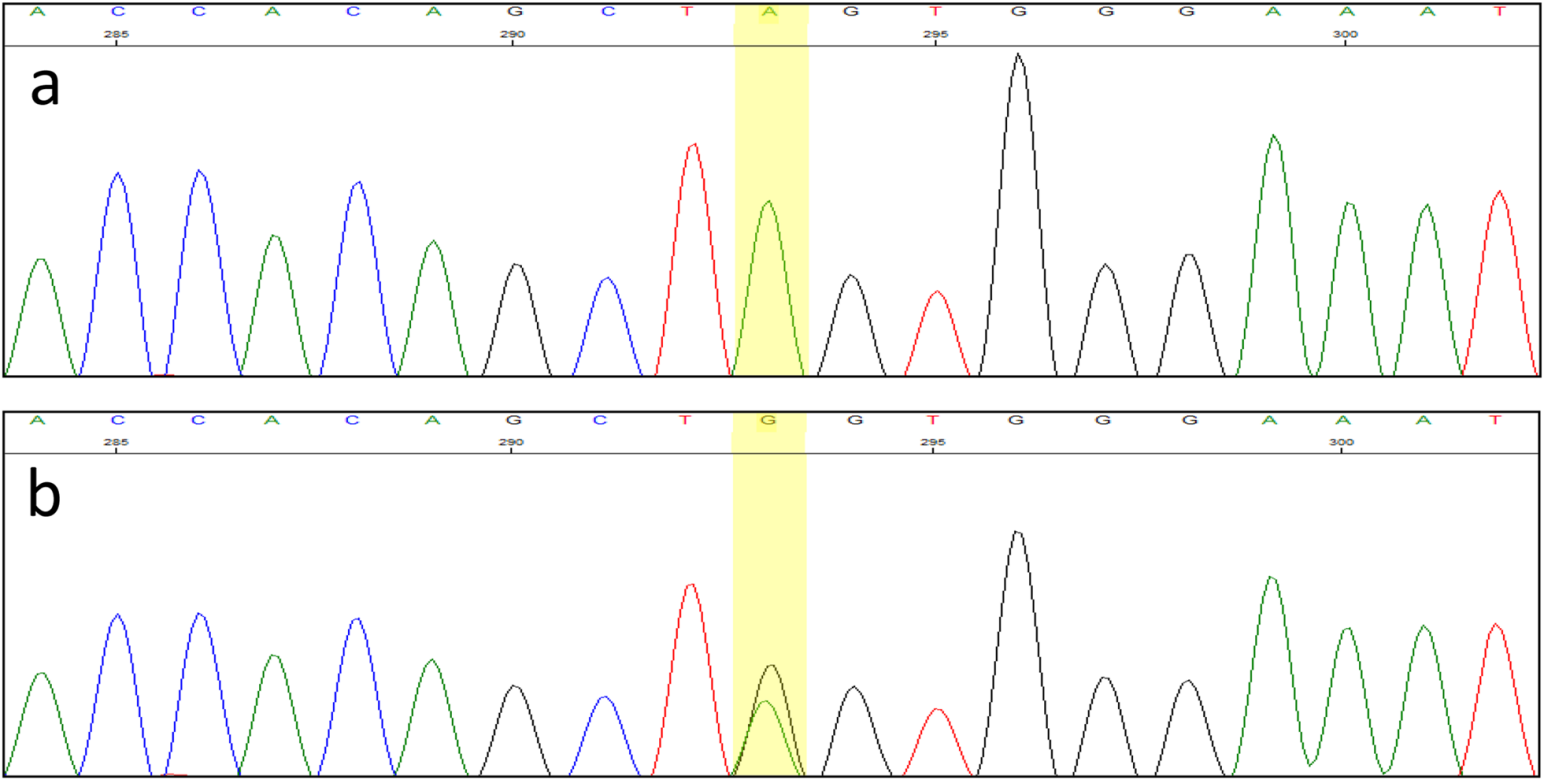
Genetic analysis by PCR-Sanger sequencing showed Hemizygosity of variant c.115G > A in IL2RG gene in the patient (**a**) and heterozygosity in his mother (**b**)

WES detected a hemizygous missense variant in exon one of the IL2RG gene (c.115 G>A, p.D39N, ChrX: 70,331,275). IL2RG gene is associated with X-linked recessive severe combined immunodeficiency (X-linked SCID). The hemizygous status of the detected variant was confirmed by PCR-Sanger sequencing in the patient (Fig. 4a). Heterozygous carrier status was also shown in his mother by PCR-Sanger sequencing (Fig. 4b). This result was consistent with the X-linked recessive pattern of inheritance.

This variant was reported previously in Human Gene Mutation Database as a disease-causing variant [12]. UniProt database has classified this variant as a ‘disease’ type variant. The variant occurred at the last nucleotide of the exon, suggesting a possible abnormal splicing event related to the change. The variant is absent in the population databases such as gnomAD, and 1000 Genome.

Several computational tools (CADD, SIFT, and PolyPhen) support the deleterious effect of this variant on gene products. Considering the above findings and based on the ACMG guideline, this variant was classified as a likely pathogenic variant. According to this variant classification, in addition to other clinical and para-clinical findings, X-linked SCID was confirmed as a diagnosis in this patient.

Another missense variant was detected in the HYDIN gene (c.2051T>C), which is classified as a Variant of Uncertain Significance. This variant is related to primary ciliary dyskinesia-5 and is inherited in an autosomal recessive pattern. However, according to the clinical phenotype and lack of such variant in the parents, it was not considered to be diagnostic.

According to the clinical presentation, paraclinical studies, and the variant detected in genomic investigations, the patient was diagnosed with X-linked SCID. Inhaled steroid, oral proton pump inhibitor, prophylactic antibiotic, and acyclovir in addition to monthly IVIG (Intravenous immunoglobulin) with a dose of 400 mg/kg were started. According to the endocrinology consult, levothyroxine consumption was continued but growth hormone was ceased.

Significant improvements in the patient’s condition were observed a few months after starting the treatment; the size of lymph nodes became smaller and gradually disappeared, fewer respiratory infections occurred, productive coughs improved. Despite prescribing the prophylactic acyclovir, herpetic lesions on lips recurred, although the intervals significantly increased, and healing became much faster than before. However, he hasn’t experienced any severe infections and was admitted to the hospital for nothing but IVIG infusion. He was referred for bone marrow transplantation, but his parents decided to continue IVIG plus prophylactic antibiotic and acyclovir.

## Discussion and conclusion

A case of X-linked SCID with a rare mutation in the IL2RG gene (c.115 G>A substitution) was described. He had been suffering from a wide variety of clinical features including allergic manifestations, growth failure, recurrent sinopulmonary infections, cervical lymphadenopathies, chronic gastrointestinal and cutaneous problems, starting from infancy.

Comprising a group of over 20 distinct genetic disorders, severe combined immunodeficiency is known as a type of PID which is featured by profound defects in both antibody production and cellular immunity. The occurrence incidence is estimated to be 1 in 50,000 to 100,000 births, though actual population incidence is believed to be more in number [13]. Different types of SCID have been reported among Iranian people and the relatively high prevalence of autosomal recessive forms of SCID is attributable to the high percentage of consanguineous marriages in this community [14, 15].

Thanks to applying the technology of genetic sequencing, pathogenic variants of over 20 genes that resulted in different SCID phenotypes, have been found. Mutations in the IL2RG gene for X-linked SCID patients were reported in 1993 for the first time [16] and since then, more than 200 mutations in the 8 exons of the IL2RG gene have been classified, most of them (119 out of 200) are frameshift mutations [14]. X-SCID patients usually present with the classic clinical phenotype. A case of atypical X-SCID was reported in 1994 presented with a high rate of infections, decreased growth rate, and persistent diarrhea. His immunological assessments unexpectedly showed that not only total levels of serum immunoglobulins but also T, B, and NK cells counts were within normal ranges. Nonetheless, specific antibody responses were not protective. Similar to the present case, a (G to A) substitution at position 115 was previously reported. However, the clinical and immunological findings were different. Even though persistent diarrhea and poor growth were found in that case, there was no report of hypothyroidism, food allergy, or herpetic lesions. Meanwhile, unlike hypergammaglobulinemia and decreased number of CD4+ T cells in the present patient, the number of T, B, and NK cells as well as serum immunoglobulin levels in the previously reported case, were within normal limits [12]. Clinically speaking, severe and persistent infections are reported to be the features of X-SCID in the first months of life, usually accompanied by the failure to thrive and diarrhea [17]. However, some patients have atypical presentations including a polymorphous lymphoproliferative disorder with Hodgkin-like features [18]. The growth failure, seen in children with X-SCID, is believed to be the result of γc subunit involvement in growth hormone-receptor signaling [19].

Several mutations in the IL2RG gene, localized in Xq13, have been identified among X-SCID patients. A transmembrane protein is coded by this gene and is recognized as a component of the IL-2 receptor. Lack of expression of gamma chain or non-functional protein can occur as a result of most of these mutations [20, 21] leading to very low T- and NK-lymphocyte counts, but the B-lymphocyte count is normal (a so-called T−, B+, NK-phenotype) [22]. Despite the high number of B-lymphocytes, there is no function since the B cells have abnormal receptors for growth factors on their cell surfaces [23].

Because of severe immune defects, a wide range of opportunistic infections can commonly be seen in SCID patients. To control infections, affected patients are prescribed appropriate antibiotic therapy and intravenous human immunoglobulin infusions until being undergone hematopoietic stem cell transplantation [24]. The same intervention was conducted on the patient in this study.

All in all, this was a report of an X-SCID patient with predominantly allergic gastrointestinal and respiratory manifestations, sluggish growth, and hypothyroidism. The patient, surprisingly, experienced no severe or life-threatening infection with any usual or opportunistic organism, similar to the previously reported case with the same mutation, which could be explained by the genotype–phenotype correlation in these patients.

Primary immunodeficiency disorders (PIDs) should be considered as the differential diagnosis of every patient with unexplained and bizarre symptoms particularly when it is associated with recurrent infection, allergic and autoimmune manifestations. Clinicians should also bear X-SCID (IL2RG gene c.115 G>A substitution) in mind in case of approach to any patient with poor weight gain, unusual allergic or endocrine manifestations. Physicians should be aware of the variable expressions of PIDs to prevent the delay in diagnosis and improve the outcome of these disorders.

## Data Availability

Not applicable.

## Abbreviations

SCID: Severe combined immunodeficiency
PIDs: Primary immunodeficiency disorders
IL-2RG: Interleukin 2 receptor
HSCT: Hematopoietic stem cell transplantation
X-SCID: X-linked SCID
IL: Interleukin
CMV: Cytomegalovirus
PR: Patient result
NR: Normal range
CT: Computed tomography
NBT: Nitroblue Tetrazolium
WES: Whole exome sequencing
BWA: Burrows-Wheeler Aligner
GATK: Genome Analysis toolkit
ACMG: American College of Medical Genetics and Genomics
HGMD: Human Gene Mutation Database
IVIG: Intravenous immunoglobulin
LTT: Lymphocyte transformation test
PHA: Phytohemagglutinin
